# Universal Nonlinear Spiking Neural P Systems with Delays and Weights on Synapses

**DOI:** 10.1155/2021/3285719

**Published:** 2021-08-25

**Authors:** Liping Wang, Xiyu Liu, Yuzhen Zhao

**Affiliations:** Business School, Shandong Normal University, Jinan, China

## Abstract

The nonlinear spiking neural P systems (NSNP systems) are new types of computation models, in which the state of neurons is represented by real numbers, and nonlinear spiking rules handle the neuron's firing. In this work, in order to improve computing performance, the weights and delays are introduced to the NSNP system, and universal nonlinear spiking neural P systems with delays and weights on synapses (NSNP-DW) are proposed. Weights are treated as multiplicative constants by which the number of spikes is increased when transiting across synapses, and delays take into account the speed at which the synapses between neurons transmit information. As a distributed parallel computing model, the Turing universality of the NSNP-DW system as number generating and accepting devices is proven. 47 and 43 neurons are sufficient for constructing two small universal NSNP-DW systems. The NSNP-DW system solving the Subset Sum problem is also presented in this work.

## 1. Introduction

Membrane computing (MC) is a representative of a new type of computing, abstracted from the phenomenon of signal transmission between cells in animals. It was proposed by Gheorghe Păun in 1998 and published in 2000 [1]. As a new type of natural computing, membrane computing has abundant model support [2–4] and is widely used in real life [5, 6]. The distributed computing model is named after the membrane system or P system. So far, P systems are mainly divided into three categories: cell-like P systems [7, 8], tissue-like P systems [9, 10], and neural-like P systems. Spiking neural P (SNP) systems [11], axon P systems [12], and dendrite P systems [13] are widely studied types of neural-like P systems. The research on SNP systems has been abundant for more than ten years. Similar to the spiking neural network (SNN), in the SNP system, neurons are activated, and the spikes are transmitted to other neurons along the synapse. SNP systems encode information through spikes in neurons. Intuitively, SNP systems are represented by a directed graph, where the nodes in the graph represent neurons, and the neurons are connected by arcs representing synapses. Neurons contain spikes and rules; two kinds of rules are used in SNP systems: firing rules (*E*/*a*^*r*^)⟶*a* and forgetting rules *a*^*s*^⟶*λ*. Generating, accepting, and functional computing are the three working modes of SNP systems. It is worth mentioning that, by introducing neuron division, budding, or dissolution, the SNP system has been proven to be able to solve some computationally difficult problems, like SAT problem and Subset Sum problem [14, 15]. SNP can also solve many practical problems such as fault diagnosis of power systems [16–18], image processing [19, 20], and combination optimization problem [21]. Based on those innovative works, more and more scholars pay attention to SNP systems. Many variants of SNP systems have been proposed, and their computing power has also been proven.

The generation of the SNP system itself is affected by the spike signal in biological phenomena. From the perspective of biological facts, Păun et al. considered another important component related to brain function, astrocytes, which implicitly control the number of spikes along the axon. The functional application of astrocytes in the SNP model was first introduced in 2007 [22]. In 2012, Pan et al. formally proposed SNP systems with astrocytes [23]. More discussions about this type of system have been formed, such as [24, 25]. For such systems, astrocytes influence computation by controlling the transmission of spikes on synapses. Considering the difference in the number of synapses connected between neurons, Pan et al. [26] previously proposed spiking neural P systems with weighted synapses. Then considering the mutual annihilation between spikes, Pan and Păun [27] used a pair of antispikes $\left(a,\bar{a}\right)$ to explain the SNP system with antispikes. In the initial SNP system, there was a unique synapse connection between two neurons. Based on biological facts, Peng et al. [28] proposed that the synapse of each neuron can have an indefinite number of channels connected to subsequent neurons, and the SNP system with multiple channels was reasonably verified. Later, for the phenomenon that neurons carry positive and negative ions, the polarized SNP system was studied [29, 30]. In view of this neurophysiological observation, the speed of information transmission on the synapse is different. Song et al. [31] proposed spiking neural P systems with delay on synapses (SNP-DS) in 2020. On the other hand, driven by the ideas of mathematics and computing science, many variants of SNP systems make the SNP computing model with a parallel mechanism more powerful, for example, a complete arithmetic calculator constructed from SNP systems [32], SNP systems with random application of rules by introducing probability [33], homogeneous SNP systems with structural plasticity by adding and deleting connections between neurons [34], SNP systems with colored spikes by the idea of colored Petri nets [35], SNP systems with communication on request by using the parallel-cooperating grammar system to communicate on request [36], SNP systems with learning functions used to identify letters and symbols [37], and numerical SNP systems inspired by the numerical nature of the numerical P (NP) systems [38].

For SNP systems and variants mentioned above, the number of spikes in neurons is in integer form. So the nonlinear spiking neural P (NSNP) systems were investigated by Peng et al. [39]. In this system, the nonnegative integer state of neurons is replaced by real numbers; the number of spikes consumed and generated is replaced by nonlinear functions. However, NSNP systems need 117 and 164 neurons to construct Turing universal systems as functional computing device and number generator, respectively. For a new heuristic computing model such as this one, computing power is an important measure. At the same time, as a computational model of exchanging space for time, the computing power of NSNP systems needs to be further improved.

In this work, for the purpose of greater calculation power of NSNP systems, inspired by [26, 31], a novel P system, the nonlinear spiking neural P systems with delays and weights on synapses are proposed, abbreviated as NSNP-DW. In NSNP-DW systems, related to several factors, such as the dendrites' length, the spikes emitted from the same firing neuron reach different postsynaptic neurons at different times. So delays on synapses are introduced into the NSNP systems. Also, the number of dendrites among a firing neuron and its postsynaptic neurons is different. Although the spikes emitted from one neuron are the same, postsynaptic neurons with different amounts of dendrites to the firing neuron receive different amounts of spikes. Therefore, weights on synapses are added to the NSNP systems, and weights are treated as multiplicative constants by which the number of spikes varies when transiting across synapses. Both of these elements play a role in enhancing the computing power of the system.

Different from NSNP systems, the main novelties of this work are as follows:

- We propose a novel P system called universal nonlinear spiking neural P systems with delays and weights on synapses closer to biological neurons.
- For the NSNP systems in [39], integer spikes are replaced by nonlinear functions. In the proposed NSNP-DW system, we find more applicable nonlinear functions that are also common in neural networks and machine learning, so as to abstract the complex responses generated by spikes.
- We prove the computational power of the new P system; in addition, 47 and 43 neurons are successfully used for simulating function calculation and number generator, respectively.
- Computational efficiency is also an important evaluation for a P system, usually considering whether the NP-complete problem can be solved in a feasible time. We prove the computational efficiency of the new P system by solving a typical NP-complete problem: Subset Sum problem in polynomial time. It is an attraction of P system solving computationally hard problems so quickly. This makes the NSNP-DW system another powerful tool to solve the NP-complete problem.

The remaining contributions of the paper are reflected as follows: Section 2 briefly shares the relevant content of register machine.  Section 3 proposes NSNP-DW systems and gives two intuitive examples. The computing power of NSNP-DW systems equivalent to the Turing machine is proven in Section 4.  Section 5 verifies the universality of the NSNP-DW system using fewer neurons. The uniform solution of the Subset Sum using the NSNP-DW system is added in Section 6. Conclusion and follow-up research on the NSNP-DW system are explained in the last section.

## 2. Prerequisites

The elements of the formal language of the SNP system can be consulted in [11, 26]. Here, we only introduce some symbolic theories used in this work, such as Turing machine construction, execution instructions, and universal Turing machine.

We show that the proposed NSNP-DW system as number generating and accepting device is equivalent to Turing machine. An arbitrary family of Turing computable natural numbers, defined as NRE, is a family of length sets for recursively enumerable languages. NRE can be characterized by a register machine *M*. The structure of the form *M*=(*m*, *H*, *l*_0_, *l*_*h*_, *I*), where *m* is the number of registers, *H* denotes the instruction tag set, *l*_0_ is the starting label, *l*_*h*_ is the halting instruction HALT, and *I* denotes the instruction set. Each instruction in *I* is one of the following three forms:

1. *l*_*i*_ : (ADD(*r*), *l*_*j*_, *l*_*k*_) (add 1 to register *r* and then execute nondeterministically the instruction with label *l*_*j*_ or *l*_*k*_).
2. *l*_*i*_ : (SUB(*r*), *l*_*j*_, *l*_*k*_) (if register *r* is nonzero, then subtract 1 from it, and go to the instruction with label *l*_*j*_; otherwise, go to the instruction with label *l*_*k*_).
3. *l*_*h*_ : HALT (the halting instruction).

By imitating the register machine, the universal NSNP-DW system was verified. When all the registers are empty, the calculation starts from *l*_0_, and the instructions in *I* are continuously executed until halting instruction *l*_*h*_. The number set generated by the register machine *M* is defined as *N*_gen_. *N*_acc_ is the number set accepted by *M*; corresponding instruction *l*_*i*_ : (ADD(*r*), *l*_*j*_, *l*_*k*_) is deterministic and can be expressed as *l*_*i*_ : (ADD(*r*), *l*_*j*_).

**Figure 1:**
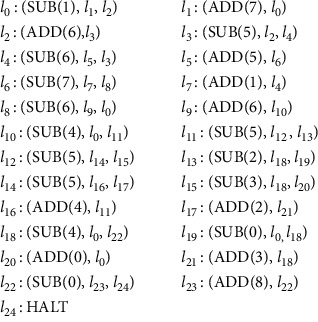
The universal register machine *M*_*u*_′.

Computable function *f* : *N*^*k*^⟶*N* (*k* is a natural number) can be calculated by the register machine. If the equation *ψ*_*x*_(*y*)=*M*_*u*_(*g*(*x*), *y*) is satisfied, where *x* and *y* are nonnegative integers and *g*(*x*) is a recursive function, then the register machine *M*_*u*_ is universal. A universal register machine *M*_*u*_′ simulated by the NSNP-DW system is shown in Figure 1, consisting of 9 registers, 14 SUB instructions, 10 ADD instructions, and one HALT instruction.

## 3. NSNP-DW Systems

The materials and methods section should contain sufficient detail so that all procedures can be repeated. It may be divided into headed subsections if several methods are described.

In the following, we provide the definition of the NSNP-DW system and related semantic explanations. An example shows the operation of the system more clearly.

### 3.1. Definition

The structure of the proposed NSNP-DW system with degree *m* ≥ 1 is

(1) $$\begin{array}{c} \Pi =\left(O,\sigma_{1},\sigma_{2},\ldots ,\sigma_{m},\text{syn},D_{\text{syn}},\text{in},\text{out}\right), \end{array}$$

where

(1) *O*={*a*} is the singleton alphabet (*a* denotes spike);
(2) *σ*_1_, *σ*_2_,…, *σ*_*m*_ are neurons, in the form of *σ*_*i*_=(*x*_*i*_, *R*_*i*_) for 1 ≤ *i* ≤ *m*, where *x*_*i*_ ∈ *R*^+^ is the initial value of spikes contained in neuron *σ*_*i*_, indicating the initial state of neuron *σ*_*i*_, and *R*_*i*_ is the finite set of spiking rules in the following form:
  - Spiking/firing rules: *E|a*^*p*(*x*_*i*_)^⟶*a*^*q*(*x*_*i*_)^, where *E* is the firing condition, *p*(*x*_*i*_) is a linear or nonlinear function, and *q*(*x*_*i*_) is a nonlinear function, and *p*(*x*_*i*_) ≥ *q*(*x*_*i*_) ≥ 0.
  - Forgetting rules: *E|a*^*p*(*x*_*i*_)^⟶*λ*, for a linear or nonlinear function *p*(*x*_*i*_).
(3) syn ∈ {1,…, *h*} × {1,…, *h*} × *W* is a synaptic expression with weight, where *W*={1,…, *n*}, *h*=*m* ∪ environment. For any (*i*, *j*, *n*) ∈ syn, 1 ≤ *i*, *j* ≤ *h*, *i* ≠ *j*, and *n* ∈ *W*.
(4) *D*_syn_ represents the delay on synapse (*i*, *j*), expressed in the form of a time number.
(5) in and out indicate the input and output neurons of the system, respectively.

The NSNP-DW system can be visualized by a directed graph *G*_Π_ with nodes and arcs, where nodes are neurons and arcs represent synapse connections. In original SNP systems, the number of spikes is described by an integer. In NSNP-DW systems, besides integer, it can also be described by nonlinear functions. For the rules *E|a*^*p*(*x*_*i*_)^⟶*a*^*q*(*x*_*i*_)^ and *E|a*^*p*(*x*_*i*_)^⟶*λ* in the NSNP-DW system, the firing condition *E* has two forms. (1) It is a regular expression like *a*(*a*^3^)^+^ to exactly “cover” the contents of the neuron. If *E* is exactly *a*^*p*(*x*_*i*_)^, then *E* can be omitted. (2) It is a threshold for enabling the rule and exists in the form of a positive real number. In order to distinguish, we write the rules as *T|a*^*p*(*x*_*i*_)^⟶*a*^*q*(*x*_*i*_)^ and *T|a*^*p*(*x*_*i*_)^⟶*λ*, *T* ∈ *R*^+^. Both types of rules can be used in this paper. We assume that *x*_*i*_(*t*) is the spike value of neuron *σ*_*i*_ at step *t*. For firing rules *T|a*^*p*(*x*_*i*_)^⟶*a*^*q*(*x*_*i*_)^, only when *x*_*i*_(*t*) ≥ *p*(*x*_*i*_) and *x*_*i*_(*t*) ≥ *T* are satisfied, the rule can be applicable. Intuitively speaking, when the firing rule is met, the neuron is fired, consuming *p*(*x*_*i*_) spikes (if the remaining (*x*_*i*_(*t*) − *p*(*x*_*i*_)) spike can no longer enable the rule, it will disappear in the neuron) and sending out *q*(*x*_*i*_) spikes. Forgetting rules *T|a*^*p*(*x*_*i*_)^⟶*λ*, *q*(*x*_*i*_) ≡ 0 mean that spikes with value *p*(*x*_*i*_) are consumed but not generated in the neuron to which it belongs. There will inevitably be multiple rules (greater than or equal to 2) in a neuron, assuming two rules, corresponding to the thresholds of *T*_1_ and *T*_2_, respectively. (i) When *T*_1_ ≠ *T*_2_ and both rules can be executed, the maximum threshold strategy is applied. For instance, if *T*_1_ > *T*_2_, the rule with *T*_1_ takes precedence, and the rule with threshold *T*_2_ is not used. For this strategy, the forgetting rule is no exception. (ii) When *T*_1_=*T*_2_=*T*, nondeterministic rule selection strategy is enabled. That is, the rules with *T*_1_ and *T*_2_ need to be discussed separately.

In addition, for the NSNP-DW system, weights and delays are reflected in synapses. For any (*i*, *j*, *n*) ∈ syn, *n* is the weight on the synapse. If the spikes with value *q*(*x*_*i*_) are emitted by neuron *σ*_*i*_, neuron *σ*_*j*_ will receive *n* × *q*(*x*_*i*_) spikes. The delay between synapses (*i*, *j*) is represented by *D*_syn_, *D*_syn_ is in the form of a time number. If there is a delay between neurons *σ*_*i*_ and *σ*_*j*_, the spiking rules *T|a*^*p*(*x*_*i*_)^⟶*a*^*q*(*x*_*i*_)^ belonging to neuron *σ*_*i*_ are enabled at step *t*. The spikes with value *p*(*x*_*i*_) are consumed from *σ*_*i*_ in step *t*+1; neuron *σ*_*j*_ will receive the spikes with value *q*(*x*_*i*_) from neuron *σ*_*i*_ at step *t*+*D*_syn_+1. Therefore, the spikes are owned by *σ*_*j*_ after *D*_syn_(*i*, *j*) moments.

Besides, the consumption function *p*(*x*_*i*_) and the production function *q*(*x*_*i*_) can be selected from the following common activation functions in neural networks:

1. Tanh function: *f*(*x*)=tanh(*x*)=(*e*^2*x*^ − 1)/(*e*^2*x*^+1).
2. Sigmoid function: *f*(*x*)=1/(1+*e*^−*cx*^).
3. RuLU function: $f\left(x\right)=\left\{\begin{array}{cc} x, & x\geq 0,\\ 0, & x<0. \end{array}\right.$
4. Derivative function of RuLU: $f\left(x\right)=\left\{\begin{array}{cc} 1, & x\geq 0,\\ 0, & x<0. \end{array}\right.$
5. ELU function: $f\left(x\right)=\left\{\begin{array}{cc} x, & x\geq 0,\\ \alpha \left(e^{x}-1\right), & x<0. \end{array}\right.$
6. $f\left(x\right)=\left\{\begin{array}{cc} 2, & x>0,\\ 0, & x\leq 0. \end{array}\right.$
7. $f\left(x\right)=\left\{\begin{array}{cc} 1, & x>0,\\ 0, & x=0,\\ -1, & x<0. \end{array}\right.$
8. $f\left(x\right)=\left\{\begin{array}{cc} 1, & x>1,\\ x, & -1\leq x\leq 1,\\ -1, & x<-1. \end{array}\right.$
9. LReLU function: $f\left(x\right)=\left\{\begin{array}{cc} x, & x>0,\\ \alpha x, & x\leq 0. \end{array}\right.$
10. PReLU function: *f*(*x*)=max(*αx*, *x*).
11. Softplus function: *f*(*x*)=log(1+*e*^*x*^).
12. Swish function: *f*(*x*)=*x* · sigmoid(*βx*).

In the NSNP-DW system, neurons are used in parallel, and the use strategy of rules in each neuron is in a sequential manner; that is, only one rule is allowed to be employed nondeterministically in a calculation step.

Assuming *m* neurons, *x*_*i*_(*t*) is the number of spikes of the *i*-th (1 ≤ *i* ≤ *m*) neuron, then the configuration (state) of the system Π at step *t* can be expressed as (*x*_1_(*t*),…, *x*_*m*_(*t*)), and the initial configuration is *X*_0_=(*x*_1_(0),…, *x*_*m*_(0)). By executing spike rules, configuration *X*_1_ to configuration *X*_2_, denoted by *X*_1_⇒*X*_2_, is defined as a transition of system Π, and the sequence obtained by this transition is defined as a calculation. Each calculation is related to a spike sequence similar to a binary sequence. The sequence is composed of 0 and 1. The output neuron emits a spike to the environment corresponding to 1; otherwise, it corresponds to 0. In this study, the time interval at which the output neuron emits spikes to the environment is used as the calculation result.

For an NSNP-DW system with at most *m* neurons and at most 2 rules in each neuron, we use N_gen_SNP_*m*_^2^ to represent all natural number sets generated by the NSNP-DW system and N_acc_SNP_*m*_^2^ to represent all natural number sets accepted by the NSNP-DW system. When the number of neurons is uncertain, *m* is often replaced by ^*∗*^.

### 3.2. Two Illustrative Examples

**Example 1 .**

**Figure 2:**
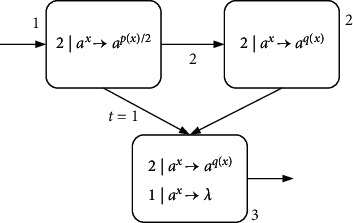
A simple example.

A simple example of the NSNP-DW system is given in Figure 2, containing three neurons labeled by 1, 2, and 3. The weight between neuron *σ*_1_ and neuron *σ*_2_ is 2. The delay between neuron *σ*_1_ and *σ*_3_ is *D*_syn_(1,3)=1, denoted by *t*=1. It is assumed here that *p*(*x*) and *q*(*x*) take (5) and (4) of the above function.

At step *t*, neuron *σ*_1_ receives two spikes from the environment, its state is *x*=2, and rule 2*|a*^*x*^⟶*a*^*p*(*x*)/2^ is applied. Neuron *σ*_1_ consumes two spikes and sends one spike to neurons *σ*_2_ and *σ*_3_ each (because (*p*(*x*)/2)=1). At step *t*+1, neuron *σ*_2_ receives two spikes due to weight 2. At this moment, neuron *σ*_3_ is not fired because of *D*_syn_(1,3)=1. At step *t*+2, neuron *σ*_3_ receives two spikes, one from neuron *σ*_1_ and one from neuron *σ*_2_ (because *q*(*x*)=1). There are two rules in neuron *σ*_3_ that both meet the fired conditions. Subject to the maximum threshold strategy, rule 2*|a*^*x*^⟶*a*^*q*(*x*)^ is used and emits one spike to the environment (for *q*(*x*)=1). This example is complete and the results of each step are presented in Table 1.

**Example 2 .**

**Figure 3:**
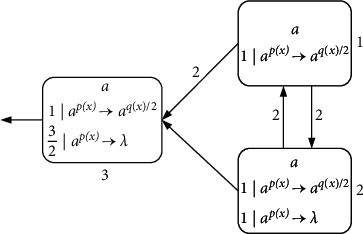
An example of simulating natural number generation.

Let *p*(*x*) and *q*(*x*) take (2) and (3) of the above functions as the consumption and generation functions. We define Π_*k*_ as the system for generating natural numbers; as shown in Figure 3, each neuron initially has a spike.

In the first step, neuron *σ*_3_ uses rule 1*|a*^*p*(*x*)^⟶*a*^*q*(*x*)/2^ to emit 1/2 spike to the environment. At the same time, neurons *σ*_1_ and *σ*_2_ also fire by applying rule 1*|a*^*p*(*x*)^⟶*a*^*q*(*x*)/2^, sending a spike to each other (because of weight 2), and neuron *σ*_1_ and neuron *σ*_2_ send one (because of weight 2) and 1/2 spike to neuron *σ*_3_, respectively. In the next step, neurons *σ*_1_ and *σ*_2_ continue their initial actions any number of times, and the 3/2 spikes in neuron *σ*_3_ are always removed. Once neuron *σ*_2_ executes rule 1*|a*^*p*(*x*)^⟶*a*^*λ*^ at step *t*, neuron *σ*_2_ stops emitting spike, and neuron *σ*_1_ sends the last spike to neuron *σ*_3_. At step *t*+1, neuron *σ*_3_ has a spike, using rule 1*|a*^*p*(*x*)^⟶*a*^*q*(*x*)/2^ to send 1/2 spike to the environment again. In this way, the time interval (*t*+1) − 1=*t* of transmitting spike to the environment, that is, the natural number *N* generated by the system Π_*k*_.

## 4. Computational Power

In the nonlinear spike rule of the NSNP-DW system, we choose two of the aforementioned functions (representing consumption or generation function) to verify the Turing universality of the system and its computing power. The following functions *p*(*x*) and *q*(*x*) are considered:

(2) $$\begin{array}{c} p\left(x\right)=\left\{\begin{array}{cc} x, & x\geq 0,\\ \alpha \left(e^{x}-1\right), & x<0, \end{array}\right.\\ q\left(x\right)=\left\{\begin{array}{cc} 1, & x\geq 0,\\ 0, & x<0. \end{array}\right. \end{array}$$

Thus, the state of neuron *σ*_*i*_ can be recorded by a nonlinear equation:

(3) $$\begin{array}{c} x_{i}\left(t+1\right)=\left\{\begin{array}{cc} x_{i}\left(t\right)-p\left(x_{i}\left(t\right)\right)+y_{i}\left(t\right), & \text{if neuron }\sigma_{i}\text{ fires,}\\ x_{i}\left(t\right)+y_{i}\left(t\right), & \text{otherwise}, \end{array}\right. \end{array}$$

where *x*_*i*_(*t*) and *x*_*i*_(*t*+1) are the states of neuron at step *t* and *t*+1, respectively, *p*(*x*_*i*_(*t*)) represents the consumption value, and *y*_*i*_(*t*) is the reception value.

In this part, we are committed to showing Turing universal NSNP-DW system as number generating device and accepting device. Given that the register machine *M* can generate and accept any NRE, the NSNP-DW system is proved to be universal through simulating the number generated by *M*. In order to facilitate understanding, we assume that number *n* in register *r* represents 2*n* spikes in neuron *σ*_*r*_. Neurons *σ*_*l*_*i*__, *σ*_*l*_*j*__, and *σ*_*l*_*k*__ receive two spikes, and the corresponding instructions are activated.

### 4.1. NSNP-DW Systems as Number Generating Device

**Theorem 1 .**

N_gen_SNP_*∗*_^2^=NRE.

**Proof**

N_gen_SNP_*∗*_^2^⊆NRE is beyond doubt based on the Turing-Church thesis; only N_gen_SNP_*∗*_^2^⊇NRE needs to be proved. In the number generating mode, *M*=(*m*, *H*, *l*_0_, *l*_*h*_, *I*) is the needed register machine, and the number generating device includes ADD, SUB, and FIN modules. *M* generates the number *n* in the following way: initially, the number of all registers is empty, and the simulation starts from instruction *l*_0_, continues the process with the required label instructions, and stops at instruction *l*_*h*_. According to the instructions, the number *n* stored in the first register is calculated by *M*. In ADD and SUB modules, neuron *σ*_*l*_*i*__ receives two spikes and runs according to instruction *l*_*i*_ : (OP(*r*), *l*_*j*_, *l*_*k*_) (OP is ADD or SUB operation). Neuron *σ*_*l*_*j*__ or *σ*_*l*_*k*__ receives two spikes indefinitely, and corresponding instruction *l*_*j*_ or *l*_*k*_ is activated. In the FIN module, neuron *σ*_out_ sends spikes outside twice at intervals.

**Figure 4:**
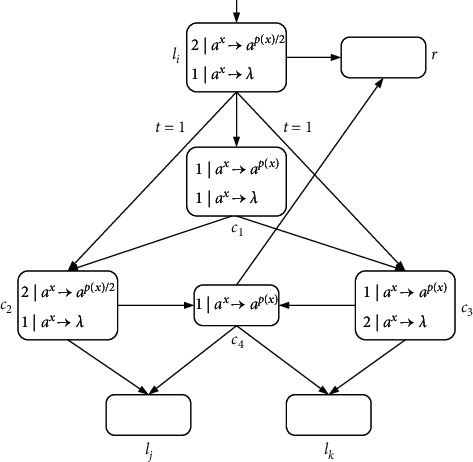
ADD module (simulating *l*_*i*_ : (ADD(*r*), *l*_*j*_, *l*_*k*_)).

ADD module (shown in Figure 4)—simulating an ADD instruction *l*_*i*_ : (ADD(*r*), *l*_*j*_, *l*_*k*_).

Neuron *σ*_*l*_*i*__ will receive two spikes from environment. After running the ADD mode, spikes will be sent to neuron *σ*_*l*_*j*__ or *σ*_*l*_*k*__ indefinitely to simulate instruction *l*_*j*_ or *l*_*k*_. When two spikes are in neuron *σ*_*r*_, the corresponding register *r* is increased by 1.

In detail, neuron *σ*_*l*_*i*__ fires at step *t*, and rule 2*|a*^*x*^⟶*a*^*p*(*x*)/2^ is executed, sending one spike to neurons *σ*_*c*_1__, *σ*_*c*_2__, *σ*_*c*_3__, and *σ*_*r*_, respectively. The next moment, neuron *σ*_*c*_1__ receives one spike. Since the same thresholds of rule 1*|a*^*x*^⟶*a*^*p*(*x*)^ and rule 1*|a*^*x*^⟶*λ*, the two rules are executed indefinitely:

- At step *t*+1, if rule 1*|a*^*x*^⟶*a*^*p*(*x*)^ is used, one spike will be sent to neurons *σ*_*c*_2__ and *σ*_*c*_3__, respectively. Next step, both neurons *σ*_*c*_2__ and *σ*_*c*_3__ contain two spikes, one from neuron *σ*_*l*_*i*__ and one from neuron *σ*_*c*_1__. At step *t*+3, neuron *σ*_*c*_3__ fires by using rule 2*|a*^*x*^⟶*λ*, so that two spikes are removed. Rule 2*|a*^*x*^⟶*a*^*p*(*x*)/2^ in neuron *σ*_*c*_2__ is applied simultaneously, consuming two spikes and emitting one to neurons *σ*_*c*_4__ and *σ*_*l*_*j*__. At the next step, neuron *σ*_*c*_4__ becomes active by executing rule 1*|a*^*x*^⟶*a*^*p*(*x*)^, emitting one spike to neuron *σ*_*r*_ and *σ*_*l*_*j*__ each. In this way, the second spike is received by *σ*_*r*_, which will aggrandize the value of register *r* by 1. Neuron *σ*_*l*_*j*__ receives a total of two spikes successively, and then instruction *l*_*j*_ starts to be simulated.
- At step *t*+1, neuron *σ*_*c*_1__ fires by using rule 1*|a*^*x*^⟶*λ*, which causes a spike to be removed. At step *t*+2, neurons *σ*_*c*_2__ and *σ*_*c*_3__ each receive one spike, and soon this no longer exists in neuron *σ*_*c*_2__ because of 1*|a*^*x*^⟶*λ*. The one received in neuron *σ*_*c*_3__ is sent to neurons *σ*_*c*_4__ and *σ*_*l*_*k*__ through rule 1*|a*^*x*^⟶*a*^*p*(*x*)^. Then, the next moment, neuron *σ*_*c*_4__ transmits one, which is received by *σ*_*r*_ and *σ*_*l*_*k*__. So neuron *σ*_*r*_ and *σ*_*l*_*k*__ have received two spikes at step *t*+4, respectively, indicating that the register *r* is increased by 1, and *l*_*k*_ is activated.

Therefore, simulating instruction *l*_*i*_ : (ADD(*r*), *l*_*j*_, *l*_*k*_) is displayed correctly. Considering two different rules, Table 2 shows the number of spikes in all neurons at each moment.

**Figure 5:**
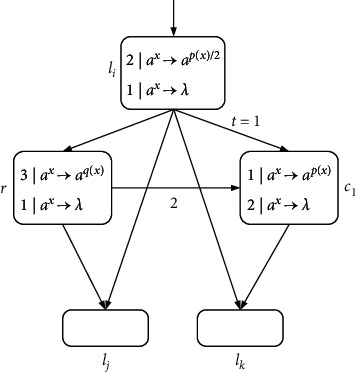
SUB module (simulating *l*_*i*_ : (SUB(*r*), *l*_*j*_, *l*_*k*_)).

SUB Module (shown in Figure 5)—simulating a SUB instruction *l*_*i*_ : (SUB(*r*), *l*_*j*_, *l*_*k*_).

Two spikes are received by neuron *σ*_*l*_*i*__. If register *r* is not empty, then two spikes are sent to neuron *σ*_*l*_*j*__, and the corresponding instruction *l*_*j*_ is executed. If the value in the register *r* is zero, then two spikes are sent to neuron *σ*_*l*_*k*__, and the instruction with label *l*_*k*_ is executed. The detailed simulation process is as follows.

Neuron *σ*_*l*_*i*__ fires at step *t*, and rule 2*|a*^*x*^⟶*a*^*p*(*x*)/2^ is applied to emit one spike. At step *t*+1, neuron *σ*_*r*_, *σ*_*l*_*j*__, and *σ*_*l*_*k*__ each receive one spike from neuron *σ*_*l*_*i*__. Next, there will be two cases according to the value of spike in neuron *σ*_*r*_:

- At step *t*+1, if 2*n*+1 (*n* ≥ 1) spikes are contained by *σ*_*r*_ (the value of the corresponding register *r* is *n*), then rule 3*|a*^*x*^⟶*a*^*q*(*x*)^ is applicable. Next step, the neuron *σ*_*c*_1__ receives three spikes and fires, one from neuron *σ*_*l*_*i*__ and two from neuron *σ*_*r*_. Based on the maximum threshold strategy, rule 2*|a*^*x*^⟶*a*^*λ*^ is used to consume these three spikes. At the same step, neuron *σ*_*l*_*j*__ receives the second spike, and then instruction *l*_*j*_ is simulated by system Π.
- At step *t*+1, if neuron *σ*_*r*_ only contains one spike (the value of the corresponding register *r* is 0), then the spike is removed by rule 1*|a*^*x*^⟶*a*^*λ*^. At step *t*+2, a spike from neuron *σ*_*l*_*i*__ is in neuron *σ*_*c*_1__. The firing of neuron *σ*_*c*_1__ by rule 1*|a*^*x*^⟶*a*^*p*(*x*)^ causes neuron *σ*_*l*_*k*__ to add a spike. At the next step, neuron *σ*_*l*_*k*__ receives a total of two spikes, and then instruction *l*_*k*_ is simulated by system Π, but not *l*_*j*_.

So SUB module simulates instruction *l*_*i*_ : (SUB(*r*), *l*_*j*_, *l*_*k*_) correctly. The simulated numerical changes are presented in Table 3.

**Figure 6:**
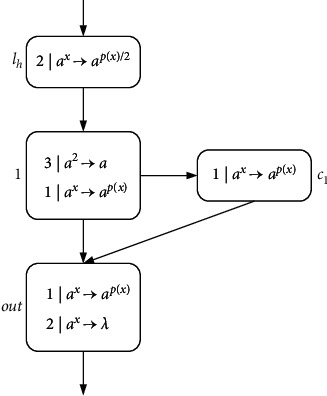
FIN Module (output calculation result).

(3) FIN module (shown in Figure 6) - outputting the result of computation.

At step *t*, neuron *σ*_*l*_*h*__ fires by running rule 2*|a*^*x*^⟶*a*^*p*(*x*)/2^, transmitting a spike to *σ*_1_. Originally, neuron *σ*_1_ contains 2(*n* − 1) (*n* ≥ 2) spikes, and after receiving one spike, the rule 3*|a*^2^⟶*a* can be used first because of the maximum threshold strategy. Then neuron *σ*_*c*_1__ and neuron *σ*_out_ each have a spike from neuron *σ*_1_. The first spike is sent by output neuron *σ*_out_ to the environment through 1*|a*^*x*^⟶*a*^*p*(*x*)^ at step *t*+3. Besides, neuron *σ*_out_ receives one spike from neuron *σ*_1_ and neuron *σ*_*c*_1__, respectively, causing them to be forgotten by 2*|a*^*x*^⟶*λ*. Since two spikes are consumed in neuron *σ*_1_, a spike is continuously given *σ*_*c*_1__ and *σ*_out_. As a result, both spikes in neuron *σ*_out_ from *t*+2 to *t*+*n* are forgotten by 2*|a*^*x*^⟶*λ*. Until step *t*+*n*+1, only one spike is kept in *σ*_1_, and then the generated one is emitted by 1*|a*^*x*^⟶*a*^*p*(*x*)^. At step *t*+*n*+2, the neuron *σ*_out_ still accepts two spikes but is forgotten instantly. At step *t*+*n*+3, the last spike is received by neuron *σ*_out_ from *σ*_*c*_1__ and sent to the environment through rule 1*|a*^*x*^⟶*a*^*p*(*x*)^. The time interval between spikes emitted to the environment is the number calculated by the system Π. In short, the numerical result computed through the system Π is (*t*+*n*+3) − (*t*+3)=*n*. Take the generated number *n*=4 as an example, and the simulated numerical changes of output module are reflected in Table 4.

Through the above discussion, we can see that the system Π accurately simulates the register machine *M*, so the theorem is reasonable.

### 4.2. NSNP-DW Systems as Number Accepting Device

**Theorem 2 .**

N_acc_SNP_*∗*_^2^=NRE.

**Proof**

**Figure 7:**
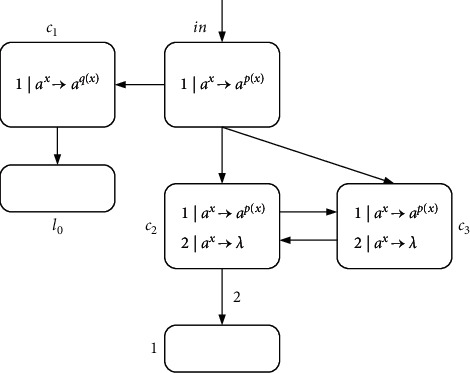
INPUT module.

In the number accepting mode, the number in the first register is *n* (others are empty), and then the calculation starts from *l*_0_; when the calculation stops, the number *n* is accepted. Similar to Theorem 1, we only need to verify N_acc_SNP_*∗*_^2^⊇NRE. The constructed NSNP-DW system as number accepting device includes an INPUT module, a deterministic ADD module, and a SUB module.  Figure 7 shows the INPUT module.

Suppose that the first spike is received by neuron *σ*_in_ at step *t*; the firing of *σ*_in_ gives a spike to neurons *σ*_*c*_1__, *σ*_*c*_2__, and *σ*_*c*_3__through rule 1*|a*^*x*^⟶*a*^*p*(*x*)^. Then, neuron *σ*_*c*_1__ fires and outputs one spike to neuron *σ*_*l*_0__, while neurons *σ*_*c*_2__ and *σ*_*c*_3__ fire by using rule 1*|a*^*x*^⟶*a*^*p*(*x*)^. At step *t*+2, neurons *σ*_*c*_2__ and *σ*_*c*_3__ send one spike to each other, and especially neuron *σ*_1_ receives two spikes from neuron *σ*_*c*_2__, until neuron *σ*_in_ receives the second spike. Thus, neuron *σ*_1_ receives 2*n* spikes from step *t*+2 to *t*+*n*+1.

At step *t*+*n*+1, neuron *σ*_in_ obtains a spike again and reacts using rule 1*|a*^*x*^⟶*a*^*p*(*x*)^, sending one spike to neurons *σ*_*c*_1__, *σ*_*c*_2__, and *σ*_*c*_3__ again. At step *t*+*n*+2, *σ*_*c*_2__ and *σ*_*c*_3__ each possess two spikes, and rule 2*|a*^*x*^⟶*λ* is applicable so that spikes are eliminated. Simultaneously, neuron *σ*_*c*_1__ fires for the second step, executing rule 1*|a*^*x*^⟶*a*^*q*(*x*)^ to give neuron *σ*_*l*_0__ a spike, whereupon instruction *l*_0_ is activated.

**Figure 8:**
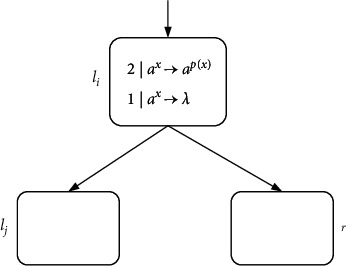
ADD module (simulating *l*_*i*_ : (ADD(*r*), *l*_*j*_)).

Figure 8 displays the simulating of deterministic ADD instruction *l*_*i*_ : (ADD(*r*), *l*_*j*_). Neuron *σ*_*l*_*i*__ accepts two spikes, consuming them and sending two spikes to neuron *σ*_*l*_*j*__ through rule 2*|a*^*x*^⟶*a*^*p*(*x*)^; instruction *l*_*j*_ is simulated. Neuron *σ*_*r*_ contains two spikes, indicating that the register *r* is increased by 1. The SUB module of accepting mode is the same as in Figure 4.

In short, N_acc_SNP_*∗*_^2^=NRE holds.

## 5. Small Universal Computing Devices

### 5.1. Small Universal NSNP-DW Systems as Function Computing Device

**Theorem 3 .**

There is a small universal NSNP-DW system possessing 47 neurons for computing functions.

**Proof**

**Figure 9:**
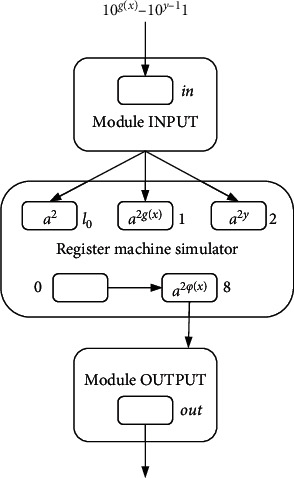
Framework of the universal NSNP-DW system.

**Figure 10:**
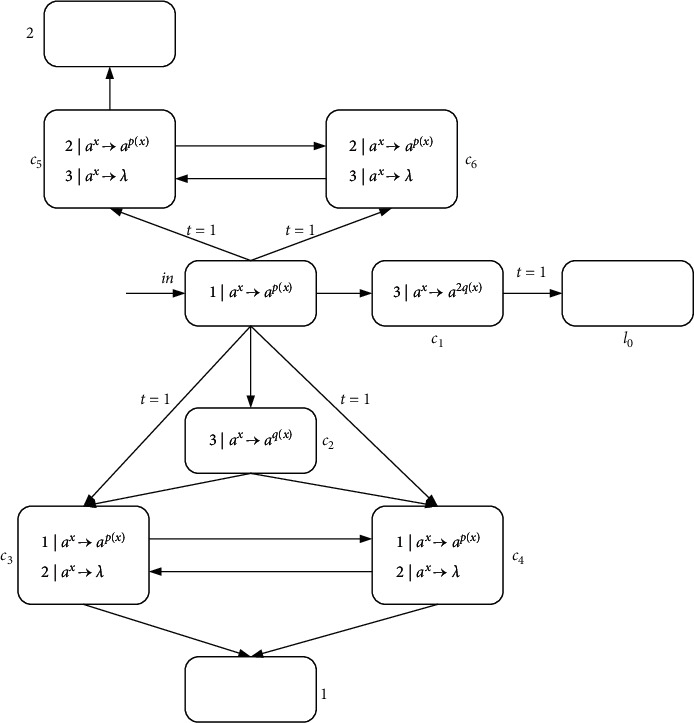
INPUT module.

For simulation of the register machine *M*_*u*_′, the designed NSNP-DW system includes INPUT, ADD, SUB, and OUTPUT modules. The general design is visualized in Figure 9. Still the same as originally assumed, the value *n* in register *r* corresponds to 2*n* spikes in neuron *σ*_*r*_. Assume that all neurons are empty in the initial configuration.  Figure 10 is the designed INPUT module. *σ*_in_ is the input neuron, reading a spike train 10^*g*(*x*)−1^0^*y*−1^1. Finally, 2*g*(*x*) and 2*y* spikes are contained by neurons *σ*_1_ and *σ*_2_, respectively.

As before, a time step or step represents the execution time of one rule. We still use this notion to mark the moment the rule is executed. At step *t*_1_, if the first spike from the environment is received by neuron *σ*_in_, rule 1*|a*^*x*^⟶*a*^*p*(*x*)^ is applicable, sending one spike to neurons *σ*_*c*_1__, *σ*_*c*_2__, *σ*_*c*_3__, *σ*_*c*_4__, *σ*_*c*_5__, and *σ*_*c*_6__, respectively. At step *t*_1_+1, neurons *σ*_*c*_3__, *σ*_*c*_4__, *σ*_*c*_5__, and *σ*_*c*_6__ will not receive spikes due to one moment of delay; neuron *σ*_*c*_1__ and neuron *σ*_*c*_2__ each receive one spike but do not fire. At the next step, the neurons *σ*_*c*_3__, *σ*_*c*_4__, *σ*_*c*_5__, and *σ*_*c*_6__ receive one spike from neuron *σ*_in_, but neurons *σ*_*c*_5__ and *σ*_*c*_6__ do not fire. Neurons *σ*_*c*_3__ and *σ*_*c*_4__ fire by employing rule 1*|a*^*x*^⟶*a*^*p*(*x*)^, sending one spike to each other and both sending one spike to *σ*_1_, so neuron *σ*_1_ owns two spikes at *t*_1_+3. In this way, neuron *σ*_1_ continues to receive two spikes, until step *t*_1_+*g*(*x*)+2, *σ*_1_ has a total of 2*g*(*x*) spikes.

At step *t*_2_, here actually *t*_2_=*t*_1_+*g*(*x*)+2, and neuron *σ*_in_ fires a second time and applies rule 1*|a*^*x*^⟶*a*^*p*(*x*)^ to send one spike to each of neurons *σ*_*c*_1__, *σ*_*c*_2__, *σ*_*c*_3__, *σ*_*c*_4__, *σ*_*c*_5__, and *σ*_*c*_6__. Neurons *σ*_*c*_1__ and *σ*_*c*_2__ each have two spikes at step *t*_2_+1. For one delay, neurons *σ*_*c*_3__, *σ*_*c*_4__, *σ*_*c*_5__, and *σ*_*c*_6__receive one spike from neuron *σ*_in_ at step *t*_2_+2. At this step, neurons *σ*_*c*_4__ and *σ*_*c*_5__ each have two spikes, and executing rule 2*|a*^*x*^⟶*λ* causes two spikes to be removed. On the contrary, neurons *σ*_*c*_5__ and *σ*_*c*_6__ each have two spikes and stay active. Two spikes are sent to each other by neurons *σ*_*c*_5__ and *σ*_*c*_6__, and two are given to *σ*_2_ by neuron *σ*_*c*_5__, so that neuron *σ*_2_ contains two spikes at step *t*_2_+3. Neuron *σ*_2_ accepts two spikes from neuron *σ*_*c*_5__ continuously until step *t*_2_+*y*+3. At step *t*_2_+*y*+3, neuron *σ*_2_ retains 2*y* spikes in total.

At step *t*_3_, here actually *t*_3_=*t*_2_+*y*+3, neuron *σ*_in_ fires a third time, one spike is consumed, and one is sent to neurons *σ*_*c*_1__, *σ*_*c*_2__, *σ*_*c*_3__, *σ*_*c*_4__, *σ*_*c*_5__, and *σ*_*c*_6__, respectively, by rule 1*|a*^*x*^⟶*a*^*p*(*x*)^. At step *t*_3_+1, neuron *σ*_*c*_1__ with three spikes fires, and the rule 3*|a*^*x*^⟶*a*^2*q*(*x*)^ is used to consume three spikes and send two spikes to neuron *σ*_*l*_0__. When the neuron *σ*_*l*_0__ receives two spikes, the introduction *l*_0_ is simulated. Neuron *σ*_*c*_2__ also fires at step *t*_3_+1 and sends one spike to neurons *σ*_*c*_3__ and *σ*_*c*_4__ through rule 3*|a*^*x*^⟶*a*^*q*(*x*)^. At step *t*_3_+2, neuron *σ*_*c*_3__ receives spikes from neuron *σ*_in_ and neuron *σ*_*c*_2__; forgetting rule 2*|a*^*x*^⟶*λ* is applied to remove two spikes. The same is true for neuron *σ*_*c*_4__. At the same step, neurons *σ*_*c*_5__ and *σ*_*c*_6__ each receive the third spike from neuron *σ*_in_, so they have three spikes and remain inactive. The forgetting rule 3*|a*^*x*^⟶*λ* is used to remove the three spikes.

In order to reflect the rationality of INPUT module, assuming *g*(*x*)=4 and *y*=3, the change in the number of spikes at each step can be clearly seen in Table 5.

**Figure 11:**
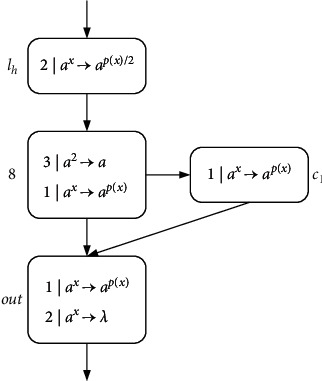
OUTPUT module.

In addition, the ADD and SUB modules are the same as in Figures 8 and 5. The design and simulation process of OUTPUT module is the same as Figure 6, except that the register 1 is replaced by register 8 (shown in Figure 11). This is because when a small universal NSNP-DW system is used as function computing device, after each instruction simulation, the final register 8 contains *n* numbers (the neuron *σ*_8_ contains 2*n* spikes). The result *n* is output through the OUTPUT module.

From the discussion above, the NSNP-DW system as a function computing device can accurately simulate the register machine *M*_*u*_′ by using 57 neurons: (i) 7 neurons in the INPUT module, (ii) 2 neurons in the OUTPUT module, (iii) 1 neuron in each SUB instruction and 14 in total, (iv) 9 neurons in 9 registers, and (v) 25 neurons for 25 instructions.

The register machine *M*_*u*_′ is simulated by the NSNP-DW system, and each instruction *l*_*i*_ on *M*_*u*_′ is regarded as a neuron. However, some instructions are continuous. By exploring the relationship between the instructions of *M*_*u*_′, correspondingly constituted modules can be combined, instructions are omitted, and the use of neurons is reduced by the way. A detailed introduction to the initial register machine and its instructions can be found in [40]. For the NSNP-DW system, module combination is mainly divided into three categories: module ADD-ADD, module ADD-SUB, and module SUB-ADD (includes modules SUB-ADD-1 and SUB-ADD-2). The working process of module ADD-SUB and module SUB-ADD is closely related to that of module SUB. The working principle is expressed by the structure diagram. Readers interested in a description of the principle can refer to [11, 26, 41].

#### 5.1.1. Module ADD-ADD

(4) $$\begin{array}{c} l_{17}:\left(\text{ADD}\left(2\right),l_{21}\right),\\ l_{21}:\left(\text{ADD}\left(3\right),l_{18}\right). \end{array}$$

**Figure 12:**
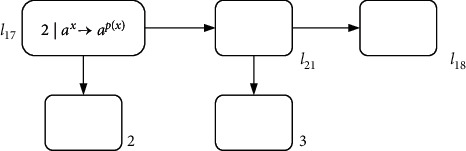
Module ADD-ADD before omitting *l*_21_: the sequence of two consecutive ADD instructions *l*_17_ : (ADD(2), *l*_21_) and *l*_21_ : (ADD(3), *l*_18_)

These are two deterministic ADD instructions. The simulation of each instruction is the same as in Figure 8. The module design is shown in Figure 12 before the instruction *l*_21_ is omitted.

**Figure 13:**
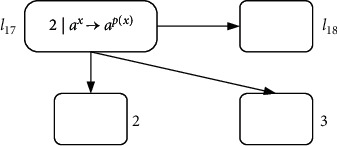
Module ADD-ADD after omitting *l*_21_: the sequence of two consecutive ADD instructions *l*_17_ : (ADD(2), *l*_21_) and *l*_21_ : (ADD(3), *l*_18_).

Obviously, this is a sequence of two consecutive ADD instructions connected by *l*_21_; instruction *l*_21_ can be omitted through the following module ADD-ADD (shown in Figure 13).

#### 5.1.2. Module ADD-SUB

(5) $$\begin{array}{c} l_{5}:\left(\text{ADD}\left(5\right),l_{6}\right),\\ l_{6}:\left(\text{SUB}\left(7\right),l_{7},l_{8}\right),\\ l_{9}:\left(\text{ADD}\left(6\right),l_{10}\right),\\ l_{10}:\left(\text{SUB}\left(4\right),l_{0},l_{11}\right). \end{array}$$

These are two consecutive pairs of ADD-SUB instructions connected by *l*_6_ and *l*_10_, respectively; we can combine instructions *l*_5_ : (ADD(5), *l*_6_) and *l*_6_ : (SUB(7), *l*_7_, *l*_8_) into one instruction *l*_5_ : (ADD(5), SUB(7), *l*_7_, *l*_8_), which saves instruction *l*_6_. Instructions *l*_9_ : (ADD(6), *l*_10_) and *l*_10_ : (SUB(4), *l*_0_, *l*_11_) are combined into one instruction *l*_9_ : (ADD(6), SUB(4), *l*_0_, *l*_11_), saving instruction *l*_10_.

**Figure 14:**
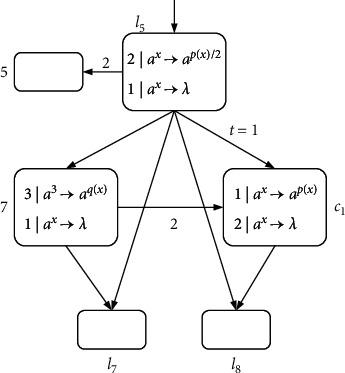
A module for consecutive ADD-SUB instruction *l*_5_ : (ADD(5), SUB(7), *l*_7_, *l*_8_).

Taking the omission of *l*_6_ as an example, neuron *σ*_*l*_5__ sends spikes to *σ*_5_ and *σ*_*l*_6__, and then neuron *σ*_*l*_6__ performs the simulation of instruction *l*_6_. Here, neuron *σ*_*l*_6__ can be omitted, and neuron *σ*_*l*_5__ replaces *σ*_*l*_6__ to directly simulate the SUB instruction. It can be seen from Figure 14 that this is possible.

#### 5.1.3. Module SUB-ADD

For introduction *l*_*i*_ : (SUB(*r*), *l*_*j*_, *l*_*k*_), when the value *r* ≠ 0, it is subtracted by 1, and the instruction *l*_*j*_ is executed; otherwise, the labeled *l*_*k*_ is activated. Therefore, considering that there are two forms of consecutive SUB-ADD instructions, module SUB-ADD is divided into modules SUB-ADD-1 and SUB-ADD-2.

Module SUB-ADD-1:

(6) $$\begin{array}{c} l_{15}:\left(\text{SUB}\left(3\right),l_{18},l_{20}\right),\\ l_{20}:\left(\text{ADD}\left(0\right),l_{0}\right). \end{array}$$

**Figure 15:**
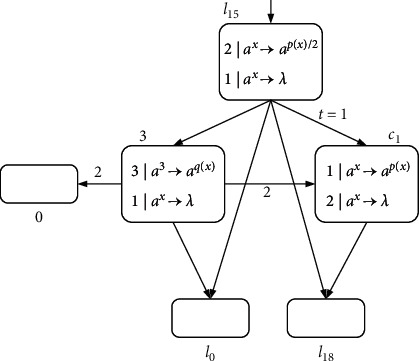
Module SUB-ADD-1: the sequence of two consecutive instructions *l*_15_ : (SUB(3), *l*_18_, *l*_20_) and *l*_20_ : (ADD(0), *l*_0_).

This is the case when *l*_*k*_ is activated and *l*_*j*_ is reserved. We can combine instructions *l*_15_ : (SUB(3), *l*_18_, *l*_20_) and *l*_20_ : (ADD(0), *l*_0_) into one instruction *l*_15_ : (SUB(3), *l*_18_, ADD(0), *l*_0_), causing instruction *l*_20_ to be omitted (see Figure 15).

Module SUB-ADD-2:

(7) $$\begin{array}{c} l_{0}:\left(\text{SUB}\left(1\right),l_{1},l_{2}\right),\\ l_{1}:\left(\text{ADD}\left(7\right),l_{0}\right),\\ l_{4}:\left(\text{SUB}\left(6\right),l_{5},l_{3}\right),\\ l_{5}:\left(\text{ADD}\left(5\right),l_{6}\right),\\ l_{6}:\left(\text{SUB}\left(7\right),l_{7},l_{8}\right),\\ l_{7}:\left(\text{ADD}\left(1\right),l_{4}\right),\\ l_{8}:\left(\text{SUB}\left(6\right),l_{9},l_{0}\right),\\ l_{9}:\left(\text{ADD}\left(6\right),l_{10}\right),\\ l_{14}:\left(\text{SUB}\left(5\right),l_{16},l_{17}\right),\\ l_{16}:\left(\text{ADD}\left(4\right),l_{11}\right),\\ l_{22}:\left(\text{SUB}\left(0\right),l_{23},l_{24}\right),\\ l_{23}:\left(\text{ADD}\left(8\right),l_{22}\right). \end{array}$$

**Figure 16:**
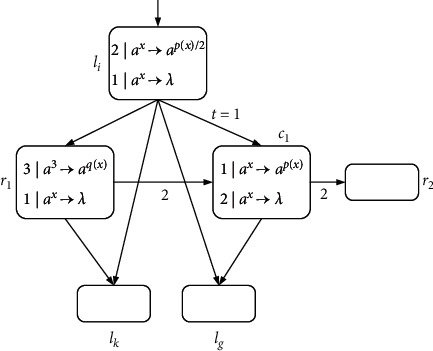
Module SUB-ADD-2: the sequence of the SUB and ADD instructions *l*_*i*_ : (SUB(*r*_1_), *l*_*j*_, *l*_*k*_) and *l*_*j*_ : (ADD(*r*_2_), *l*_*g*_).

This is the case when *l*_*j*_ is activated and *l*_*k*_ is reserved. There are six pairs of instructions that can be combined in pairs. It is found through observation that the following ADD instruction happens to be the first execution position of the previous SUB instruction. Then each pair of SUB-ADD instruction combinations can be updated to *l*_*i*_ : (SUB(*r*_1_), *l*_*j*_, *l*_*k*_) and*l*_*j*_ : (ADD(*r*_2_), *l*_*g*_). When the register *r*_1_ ≠ *r*_2_, they can share one neuron *σ*_*l*_*j*__. In this way, six neurons in total of *σ*_1_, *σ*_5_, *σ*_7_, *σ*_9_, *σ*_19_, and *σ*_23_ can be saved. The visualization can be illustrated in Figure 16.

Through the above instruction combination (called “code optimization” in [41]), 10 neurons *σ*_21_, *σ*_6_, *σ*_10_, *σ*_20_, *σ*_1_, *σ*_5_, *σ*_7_, *σ*_9_, *σ*_16_, and *σ*_23_ can be omitted. In the end, 47 neurons are enough to complete a small universal NSNP-DW system for computing functions:

- Nine neurons for 9 registers.
- 15 neurons in remaining 15 instruction labels (ten labels are saved).
- Seven neurons in the module INPUT.
- 14 neurons for a total of 14 SUB instructions.
- Two neurons in the module OUTPUT.

### 5.2. Small Universal NSNP-DW Systems as Number Generator

**Figure 17:**
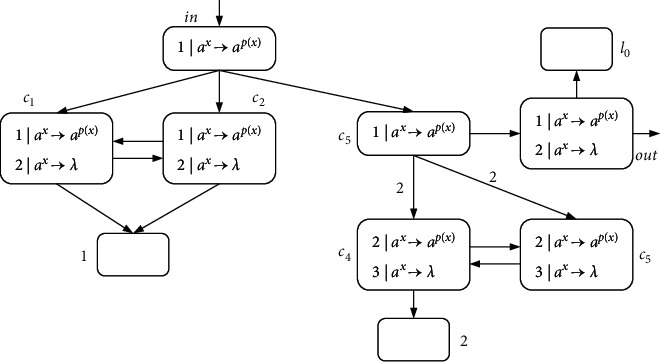
INPUT-OUTPUT module.

In the simulation of number generator, the INPUT module can be combined with the OUTPUT module, found in Figure 17. In the constructed INPUT-OUTPUT module, instruction *l*_*h*_ is removed, and register 8 is no longer needed. The spike train that the input neuron gets from the environment is 10^*g*(*x*)−1^1, neuron *σ*_1_ is loaded with 2*g*(*x*) spikes, and neuron *σ*_2_ receives 2*n* spikes nondeterministically. Neuron *σ*_out_ fires twice successively, and the time interval *n* is the numerical result generated.

The simulation of NSNP-DW systems as number generator shares 43 neurons, and the specific details will not be repeated:

- Eight neurons for 8 registers (no register 8).
- 14 neurons in the remaining 14 instruction labels (*l*_*h*_ and ten labels are saved).
- Seven neurons in the module INPUT-OUTPUT.
- 14 neurons for a total of 14 SUB instructions.

**Theorem 4 .**

There is a small universal NSNP-DW system possessing 43 neurons for number generator.

The specific simulation will not be introduced in detail. We use an example to analyze the feasibility of number generator simulation; assuming *g*(*x*)=2 and *n*=2, the results of each step are reflected in Table 6.

### 5.3. Discussion

Theorem 3 gives the Turing universal NSNP-DW system with fewer neurons as a function computing device. In order to more intuitively verify the computing power of the NSNP-DW system, Table 7 compares the number of computing units for the variant and its related systems. According to Table 7, we observe that NSNP systems, SNP systems, SNP-DS systems, and recurrent neural networks use 117, 67, 56, and 886 neurons, respectively, to accomplish Turing universality for computing function, and NSNP-DW systems only require 47 neurons. Besides, according to Table 8, we have observed that 121 neurons are reduced for simulating number generator. In short, NSNP-DW systems are better than these systems in the use of neurons, and the computational power of the NSNP system has been effectively improved.

## 6. A Uniform Solution to Subset Sum Problem

The Subset Sum problem is one of the typical NP-complete problems proposed in [43]. We use the NSNP-DW system to solve the uniform solution of the Subset Sum in a nondeterministic operation mode.

The Swish function and the LReLU function are considered for the spike consumption function and the generating function in the problem.

(8) $$\begin{array}{c} \phi \left(x\right)=x\cdot \text{sigmoid}\left(x\right),\\ \gamma \left(x\right)=\left\{\begin{array}{cc} x, & x>0,\\ \alpha x, & x\leq 0. \end{array}\right. \end{array}$$

Problem. NAME: Subset Sum.

INSTANCE: a set of positive integers *V*={*v*_1_, *v*_2_,…, *v*_*n*_} and a positive integer *S*.

QUESTION: is there a subset *B*⊆*V* that satisfies ∑_*b*∈*B*_*b*=*S*?

**Theorem 5 .**

The uniform solution of Subset Sum problem can be solved by NSNP-DW systems.

**Figure 18:**
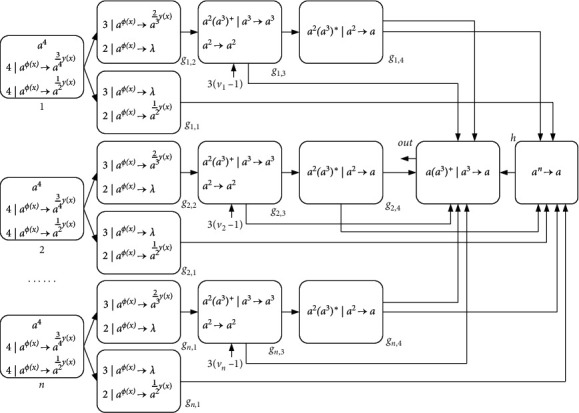
An NSNP-DW system solving the Subset Sum problem.

Figure 18 depicts the architecture of the NSNP-DW system to solve the Subset Sum in a uniform way. The complexity of the uniform solution is that the system only “recognizes” the number *n* when solving the problem. The instance of the problem needs to be introduced into the system. *σ*_*g*_*i*,3__ (1 ≤ *i* ≤ *n*) is the input neuron of the system. The positive integer *v*_*i*_ (1 ≤ *i* ≤ *n*) in the problem is encoded into *σ*_*g*_*i*,3__. At the beginning of the calculation, 3(*v*_1_ − 1) spikes (*a*^3(*v*_1_ − 1)^) enter neuron *σ*_*g*_1,3__, 3(*v*_2_ − 1) spikes (*a*^3(*v*_2_ − 1)^) enter neuron *σ*_*g*_2,3__, ..., and 3(*v*_*n*_ − 1) spikes (*a*^3(*v*_*n*_ − 1)^) enter neuron *σ*_*g*_*n*,3__. In the initial configuration (state) of the system, except that neuron *σ*_*i*_ (1 ≤ *i* ≤ *n*) contains four spikes, all other neurons are empty.

In the first calculation, both rules in neuron *σ*_*i*_ are likely to be employed first (because they have the same threshold). The indeterminate use of these two rules indicates that the system solves this Subset Sum problem in a nondeterministic way of operation, and it also corresponds to whether the integer *v*_*i*_ is in the subset *B*. In the following, we carry out a complete derivation.

**Proof**

Neuron *σ*_*i*_ initially has four spikes. At step one, if rule 4*|a*^*ϕ*(*x*)^⟶*a*^(3/4)*γ*(*x*)^ is selected for use, then neuron *σ*_*i*_ will consume 4 · sigmoid(4) spikes and send three spikes to neurons *σ*_*g*_*i*,1__ and *σ*_*g*_*i*,2__, respectively (because *γ*(*x*)=4). At step 2, neuron *σ*_*g*_*i*,1__ forgets three spikes by the rule 3*|a*^*ϕ*(*x*)^⟶*λ*. At the same time, neuron *σ*_*g*_*i*,2__ uses rule 3*|a*^*ϕ*(*x*)^⟶*a*^(2/3)*γ*(*x*)^ to become active and sends two spikes to neurons *σ*_*g*_*i*,3__ (because *γ*(*x*)=3). At the end of this step, neurons *σ*_*g*_*i*,1__ and *σ*_*g*_*i*,2__ maintain their original state. At step 3, neuron *σ*_*g*_*i*,3__ has a total of 3*v*_*i*_ − 1 spikes and fires. The rule *a*^2^(*a*^3^)^+^*|a*^3^⟶*a*^3^ is used first, sending three spikes to neurons *σ*_*g*_*i*,4__ and *σ*_out_, respectively. This process will continue for *v*_*i*_ − 1 steps until the rule *a*^2^(*a*^3^)^+^*|a*^3^⟶*a*^3^cannot be activated. Neurons *σ*_*g*_*i*,4__ and *σ*_out_ still cannot be active after receiving 3*k* (*k* ∈ *N*) spikes. When there are only two spikes left in neuron *σ*_*g*_*i*,3__, rule *a*^2^⟶*a*^2^ is used, and finally, two spikes are sent to neuron *σ*_*g*_*i*,4__ and *σ*_out_, respectively. After possessing 3*k*+2 spikes, neuron *σ*_*g*_*i*,4__ fires and emits a spike to neurons *σ*_out_ and *σ*_*h*_, respectively. In the next step, the neuron *σ*_out_ still cannot fire because it takes 3*k*+1 spikes to be activated. Conversely, neuron *σ*_*h*_ that has received *n* spikes is activated by rule *a*^*n*^⟶*a* and sends one spike to neuron *σ*_out_. In this way, the output neuron *σ*_out_ can fire and emit spikes to the environment.

If initially neuron *σ*_*i*_ uses the rule 4*|a*^*ϕ*(*x*)^⟶*a*^(1/2)*γ*(*x*)^, 4 · sigmoid(4) spikes are consumed and two spikes are sent to neurons *σ*_*g*_*i*,1__ and *σ*_*g*_*i*,2__, respectively (because *γ*(*x*)=4). In the second step, the two spikes received by neuron *σ*_*g*_*i*,2__ are removed by rule 2*|a*^*ϕ*(*x*)^⟶*λ*. Neuron *σ*_*g*_*i*,1__ uses the rule 2*|a*^*ϕ*(*x*)^⟶*a*^(1/2)*γ*(*x*)^ and sends a spike to neuron *σ*_*h*_ (because *γ*(*x*)=2). Before the neuron *σ*_*h*_ fires, neuron *σ*_out_ remains inactive. After neuron *σ*_*h*_ receives a total of *n* spikes from *σ*_*g*_*i*,1__ (1 ≤ *i* ≤ *n*), it fires and sends a spike to neuron *σ*_out_. In the next step, the neuron *σ*_out_ contains only one spike, so it does not fire, nor does it emit spikes into the environment.

At this point, we have ended the process of solving the uniform solution of the Subset Sum. Obviously, the system requires a total of 5*n*+2 neurons. After stopping operation, if there are exactly *S* spikes in the environment, the answer to the problem is “yes,” which means that there is a subset *B*⊆*V* that makes ∑_*b*∈*B*_*b*=*S* hold. Otherwise, it is “no.” This is enough to show that the NSNP-DW system for solving Subset Sum problem is complete.

In the calculation process, the calculation between neurons is parallel, and the rules in each neuron are calculated sequentially. Through computing and reasoning, it can be known that neurons *σ*_*i*_, *σ*_*g*_*i*,1__, *σ*_*g*_*i*,2__, *σ*_*g*_*i*,4__, and *σ*_*h*_ fire once, respectively, and neuron *σ*_*g*_*i*,3__ fires ∑_*i*=1_^*n*^*v*_*i*_ times. After all other neurons stop computing, the neuron *σ*_out_ can fire at most ∑_*i*=1_^*n*^*v*_*i*_ times. Therefore, the system needs ∑_*i*=1_^*n*^2*v*_*i*_+5 steps to complete the calculation. In addition, we choose nonlinear functions as the spike consumption function and generation function, which is closer to reality and reflects the significance of nonlinear functions in the NSNP-DW system.

## 7. Conclusions and Further Work

The nonlinear spiking neural P (NSNP) systems are variants of spiking neural P (SNP) systems. Nonlinear functions are used flexibly in NSNP systems. We focus on the computing power of NSNP systems in this work. Two mechanisms of delays and weights are introduced, and nonlinear spiking neural P systems with delays and weights (NSNP-DW) are proposed. An explicit example is given to visually demonstrate the operation of the NSNP-DW system. Through a series of simulation computing, 47 and 43 neurons are sufficient for constructing small universal NSNP-DW systems as function computing device and number generator. Compared with the NSNP systems [39], the NSNP-DW system decreases 70 neurons and 121 neurons, respectively, as function computing device and number generator. Finally, the uniform solution of the Subset Sum problem is solved efficiently by using the NSNP-DW system.

For further work, the NSNP-DW system, as a distributed parallel computing model, can be combined with clustering algorithms to improve algorithm efficiency. As far as the computational power of the NSNP-DW system is concerned, we are committed to proving that the 47 and 43 neurons used by the simulating function calculation and the number generator, respectively, are the least in total. In particular, the number of spikes breaks through the integer limit and has been replaced by nonlinear functions in NSNP systems. In view of this breakthrough, we can try to link NSNP-DW systems with the neural network to expand more interesting research.

## Figures and Tables

**Table 1:** Number of spikes at each moment in neurons.

Step	*σ*_1_	*σ*_2_	*σ*_3_
*t*	2	0	0
*t*+1	0	2	0
*t*+2	0	0	2 (fire)

**Table 2:** The spike result under ADD module.

Step	*σ*_*l*_*i*__		*σ*_*c*_1__		*σ*_*c*_2__		*σ*_*c*_3__		*σ*_*c*_4__		*σ*_*l*_*j*__		*σ*_*l*_*k*__				*σ*_*r*_
*t*	2		0		0		0		0		0		0				2*n*
*t*+1	0		1		0		0		0		0		0				2*n* + 1

	If 1*|a*^*x*^⟶*a*^*p*(*x*)^ is used								If 1*|a*^*x*^⟶*λ* is used								

	*σ*_*l*_*i*__	*σ*_*c*_1__	*σ*_*c*_2__	*σ*_*c*_3__	*σ*_*c*_4__	*σ*_*l*_*j*__	*σ*_*l*_*k*__	*σ*_*r*_	*σ*_*l*_*i*__	*σ*_*c*_1__	*σ*_*c*_2__	*σ*_*c*_3__	*σ*_*c*_4__	*σ*_*l*_*j*__	*σ*_*l*_*k*__	*σ*_*r*_	

*t*+2	0	0	2	2	0	0	0	2*n* + 1	0	0	2	2	0	0	0	2*n* + 1	
*t*+3	0	0	0	0	1	1	0	2*n* + 2	0	0	0	0	1	0	1	2*n* + 1	
*t*+4	0	0	0	0	0	2	1	2*n* + 1	0	0	0	0	0	1	2	2*n* + 2	
*t*+5	0	0	0	0	0	2	0	2*n* + 1	0	0	0	0	0	0	2	2*n* + 2	

**Table 3:** The results of spike in SUB module.

	*σ*_*r*_ is not empty					*σ*_*r*_ is empty				
Step	*σ*_*l*_*i*__	*σ*_*c*_1__	*σ*_*l*_*j*__	*σ*_*l*_*k*__	*σ*_*r*_	*σ*_*l*_*i*__	*σ*_*c*_1__	*σ*_*l*_*j*__	*σ*_*l*_*k*__	*σ*_*r*_
*t*	2	0	0	0	2*n* + 1	2	0	0	0	0
*t*+1	0	0	1	1	2*n* + 2	0	0	1	1	1
*t*+2	0	3	2	1	2*n* − 1	0	1	1	1	0
*t*+3	0	0	2	1	2*n* − 1	0	0	1	2	0
*t*+4	0	0	2	0	2*n* − 1	0	0	0	2	0

**Table 4:** The results of spike in FIN module.

Step	*σ*_*l*_*h*__	*σ*_*c*_1__	*σ*_1_	*σ*_out_	Environment
*t*	2	0	6	0	0
*t*+1	0	0	7	0	0
*t*+2	0	1	5	1	0
*t*+3	0	1	3	2	1
*t*+4	0	1	1	2	0
*t*+5	0	0	0	2	0
*t*+6	0	0	0	1	0
*t*+7	0	0	0	0	1

**Table 5:** The results of spike in INPUT module when *g*(*x*)=4 and *y*=3.

Step	*σ*_in_	*σ*_*c*_1__	*σ*_*c*_2__	*σ*_*c*_3__	*σ*_*c*_4__	*σ*_*c*_5__	*σ*_*c*_6__	*σ*_1_	*σ*_2_	*σ*_*l*_0__
*t*	1	0	0	0	0	0	0	0	0	0
*t*+1	0	1	1	0	0	0	0	0	0	0
*t*+2	0	1	1	1	1	1	1	0	0	0
*t*+3	0	1	1	1	1	1	1	2	0	0
*t*+4	1	1	1	1	1	1	1	4	0	0
*t*+5	0	2	2	1	1	1	1	6	0	0
*t*+6	0	2	2	2	2	2	2	8	0	0
*t*+7	1	2	2	0	0	2	2	8	2	0
*t*+8	0	3	3	0	0	2	2	8	4	0
*t*+9	0	0	0	2	2	3	3	8	6	0
*t*+10	0	0	0	0	0	0	0	8	6	2

**Table 6:** The results of spike in INPUT-OUTPUT module.

Step	*σ*_in_	*σ*_*c*_1__	*σ*_*c*_2__	*σ*_*c*_3__	*σ*_*c*_4__	*σ*_*c*_5__	*σ*_1_	*σ*_2_	*σ*_out_	*σ*_*l*_0__
*t*	1	0	0	0	0	0	0	0	0	0
*t*+1	0	1	1	1	0	0	0	0	0	0
*t*+2	1	1	1	0	2	2	2	0	1 (fire)	0
*t*+3	1	2	2	1	2	2	4	2	0	1
*t*+4	0	0	0	0	4	4	4	4	1 (fire)	1
*t*+5	0	0	0	0	0	0	4	4	0	2

**Table 7:** Comparison of the least neurons of several calculation models.

Computing models	Number of neurons
NSNP-DW systems	47
NSNP systems [39]	117
SNP systems [11]	67
SNP-DS systems [31]	56
Recurrent neural networks [42]	886

**Table 8:** Comparison of the least neurons as number generator.

Computing models	Number of neurons
NSNP-DW systems	43
NSNP systems [39]	164

## Data Availability

No datasets were used in this article.
